# Tunable electrochemistry and efficient antibacterial activity of plant-mediated copper oxide nanoparticles synthesized by *Annona squamosa* seed extract for agricultural utility†

**DOI:** 10.1039/d1ra02382a

**Published:** 2021-05-19

**Authors:** Pooja Singh, Kshitij RB Singh, Jay Singh, Subha Narayan Das, Ravindra Pratap Singh

**Affiliations:** Department of Biotechnology, Indira Gandhi National Tribal University Amarkantak M.P. 484887 India rpsnpl69@gmail.com ravindra.singh@igntu.ac.in +91-91-0934-6565; Department of Chemistry, Govt. V. Y. T. PG Autonomous College Durg Chhattisgarh India; Department of Chemistry, Institute of Science, Banaras Hindu University Varanasi U.P. 221005 India; Department of Botany, Indira Gandhi National Tribal University Amarkantak M.P. 484887 India

## Abstract

The present report deals with the synthesis of plant-mediated copper oxide nanoparticles (pm-CuO NPs) from *Annona squamosa* aqueous seed extract for effective antibacterial activity and the further utilization of this as a platform for the electrocatalytic determination of hydrogen peroxide (H_2_O_2_) for applications in the agricultural domain. The structural, optical and morphological characteristics of the synthesized pm-CuO NPs were analyzed by UV-Vis, XRD, FT-IR, AFM, SEM, TEM, HR-TEM and EDX. After this, pm-CuO NPs were preliminarily investigated for antibacterial activity on Gram-positive and Gram-negative bacterial strains, and further, their activity was validated for assessing their antibacterial efficiency on the *Xanthomonas oryzae*, a plant pathogenic bacteria strain, and the obtained results showed that pm-CuO NPs have potency as an effective antibacterial agent for the treatment of the bacterial blight of rice caused by *X. oryzae* in the rice crop, which reduces the rice crop productivity. Further, pm-CuO NPs were electrophoretically deposited onto an indium–tin–oxide (ITO) glass substrate and assessed for the electro-oxidation of H_2_O_2_ by cyclic voltammetry (CV), and from this it was proved that pm-CuO NPs had a very high electrochemical sensitivity of 49 μA μM^−1^ cm^−2^ towards H_2_O_2_ and a low detection limit of 574 μM, with these responses obtained under optimized experimental conditions. Thus, pm-CuO NPs also provide a potential sensing platform for electrochemical studies to detect H_2_O_2_ produced during plant stress surroundings to properly manage crops susceptible to oxidative damage by elevated H_2_O_2_ levels during stress.

## Abstract 2

This study reports plant mediated-copper oxide nanoparticles synthesis from Annona squamosa and their real-life application in agricultural domain for label-free detection of H_2_O_2_ and its antibacterial efficiency for combating rice crop pathogen.

## Graphical abstract

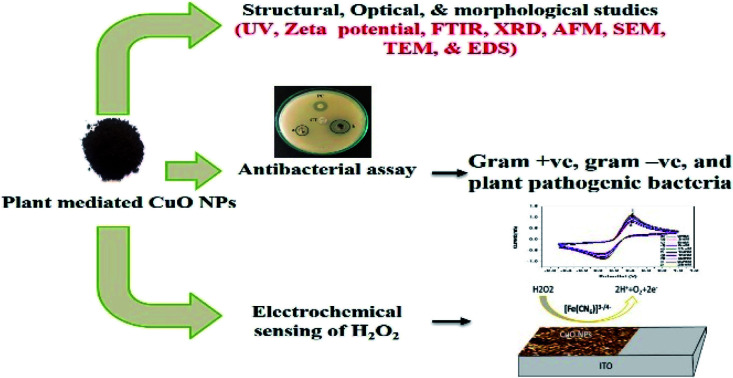

## 1. Introduction

Over the past decade, there have been interdisciplinary fields of nanoscience and nanotechnology, interspersing material science, bionanosciences, and biotechnology. The synthesis of metal oxide nanoparticles is an important research subject in modern physics due to their extraordinary capabilities in the fields of electronics, magnetics, optoelectronics, information storage and drug delivery.^1–4^ Nanoparticles of different shapes and sizes are subject to great interest due to their possible applications in industry, biomedical diagnostics, environmental remediation and electronics. Both metal and metal oxide nanoparticles have a large surface volume ratio and are considered most promising and remarkable agents in the agricultural domain. Recently, copper (Cu) and copper oxide nanoparticle have shown various applications in addressing physical, chemical and environmental issues and have also provided various solutions for solving problems pertaining to biological sciences. Chemical and physical methods can be used to synthesize nanoparticles, but they tend to be expensive, harmful to the environment, and need high energy consumption. Thus, routes involving a biological synthesis utilizing various extracts of plants, enzymes or microorganisms, including bacteria, and fungi, have been suggested as possible environment-friendly alternative methods over chemical and physical methods.^5–8^ Moreover, the synthesis of nanoparticles utilizing plant extracts is more beneficial than other biological processes as plant organs are more stable, environmentally friendly, show a higher yield of nanoparticles, and are faster than the single-step biosynthesis process.^9^ The plant extracts consist of various flavonoids, alkaloids and proteins, which help in the non-agglomeration and stabilization of nanoparticles.^10–12^ It has also been observed that some plants readily uptake and reduce metal ions from salt or mineral-rich soil by a process of detoxification and can convert them into nanoparticles.^5,13^ Numerous reports are available on the plant-mediated synthesis of metallic nanoparticles, notably from *Azadirachta indica*, *Ocimum sanctum*, *Magnolia cobus*, *Mangrera indica*, *Pomegranate*, *Calotropis gigantine*, *Annona squamosa* and extracts of coriander seeds. Indeed, the plant-mediated synthesis of nanoparticles and their aspects have gained a lot of attention nowadays due to the unique properties exhibited by nanoparticles synthesized from this route.^14–16^ Moreover, biogenic metal/metal oxide-based nanoparticles have a wide range of utility in various fields, and in agriculture, they are widely used as an antimicrobial agent, for monitoring the plant stress condition and for the detection of effluents (pesticides, by-products in pesticides (hydrazine), *etc.*) produced from erroneous agricultural practices.^17–19^

**Fig. 1:**
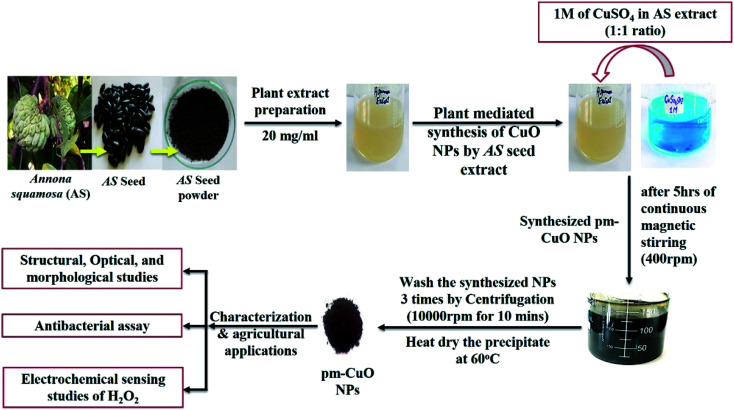
Complete overview of this work by schematic representation.

*Annona squamosa* belongs to the family of Annonaceae and is also known as custard apple. It is considered an important plant for biomedical purposes as it contains many essential elements, like potassium, calcium, zinc, selenium, magnesium, copper and phosphorous. It is also rich in carbonate, esters, terpenes, alcohol, α-pinene, sabinene and limonene; these phytochemical constituents are important in the therapy of various diseases, like vision correction, building immunity, increasing haemoglobin, equalizing the water balance in the body, *etc.*^20^ copper/cupric oxide has excellent physiochemical features, such as antibacterial, antioxidant, low-cost and is non-toxic, which make it an affordable and valuable material with enormous utility and it is also a very effective semiconductor material. Copper and copper oxide nanoparticles have promising usages as water remediation agents, sensors, photocatalysts, *etc.* Many biological synthesis approaches have been used to produce copper oxide nanoparticles, but green approaches are considered to exhibit better antibacterial and electrochemical properties. It has been observed that green synthesized copper oxide nanoparticles exhibit potential antibacterial properties when observed against various bacterial strains. Similarly, they have also shown good antioxidant, electrochemical, optical and catalytic properties. These properties have led to copper oxide nanoparticles attracting interest for many potential applications, like antimicrobial activity, electrochemical sensing, *etc.* Further, copper oxide nanoparticles have also been utilized to detect various chemicals, like hydrogen peroxide (H_2_O_2_), ammonia, glucose and lactic acid, *etc.*^21^ Herein in this study (Fig. 1), we report the synthesis of plant mediated-copper oxide nanoparticles (pm-CuO NPs) from *Annona squamosa* (AS) aqueous seed extract (see ESI file Section 1.1 to 1.3†) and their real-life application in the agricultural domain for two applications: the label-free detection of H_2_O_2_ to monitor the higher level of H_2_O_2_ concentrations in plants, which can help prevent plant death due to oxidative damage, and their antibacterial efficiency for combating the rice crop pathogen.

## 2. Results and discussion

### 2.1. Structural, optical and morphological characterization of the pm-CuO NPs

**Fig. 2:**
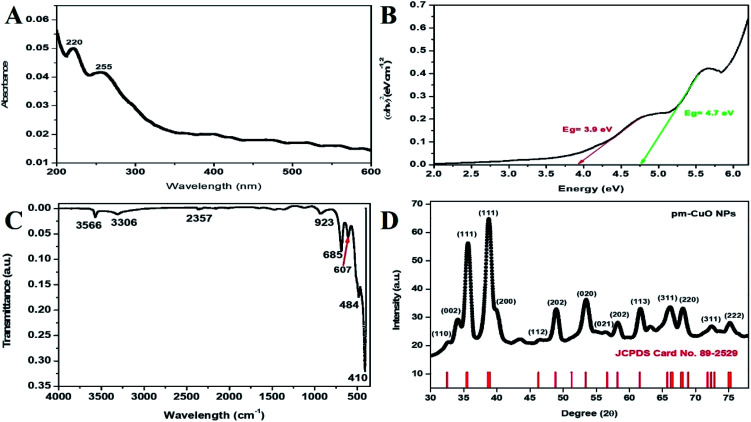
(A) Absorption spectra for UV-vis analysis, (B) Tauc plot demonstrating the energy band gaps of pm-CuO NPs, (C) FTIR vibration spectra of pm-CuO NPs and (D) XRD pattern of pm-CuO NPs.

Initially, the synthesized pm-CuO NPs were analyzed with a UV-vis spectrophotometer between 200–600 nm scan range. Nanoparticles in the solvent were excited by light absorption at different wavelengths to give respective broad peaks owing to surface plasmon resonance, which represented the reduction of the metal oxide NPs. These NPs showed a broad peak at 220 and 255 nm (Fig. 2A), corresponding to copper oxide, and these peaks were in agreement with the previously reported literature,^22,23^ and from this, it was confirmed that the synthesized nanoparticles were pm-CuO NPs. Further, the Tauc plot was used to calculate the energy band gap, as shown in Fig. 2B. The measured bandgaps were 3.9 and 4.7 eV, corresponding to two absorption bands that had been calculated by plotting the graph between energy *vs.* (*αhν*)^2^ (eV cm^−1^)^2^, where *α* is the absorption coefficient, *ν* is the frequency of the incident rays, and *h* is Plank's constant. Moreover, from this, it could also be concluded that the copper sulphate pentahydrate, when added in AS aqueous seed extract, led to the observed colour change from bluish to dark brown due to its reduction to pm-CuO NPs; here, the plant extract acted as a reducing agent as the plant extract is rich in phytochemical constituents.

FTIR study of the pm-CuO NPs was performed against the wavenumber *vs.* transmittance to analyze the synthesized pm-CuO NPs within the scan range of 4000–500 cm^−1^ to determine the phytochemicals responsible for the stabilization, capping and formation. Here, the bioactive phytochemicals present in the plant extract act as stabilizing and capping agents that provide a particular shape, size and help avoid agglomeration between the particles.^24^ As observed in Fig. 2C, the strong peak at 3356 cm^−1^ corresponded to the phenolic compound present in the AS seed extract with –OH stretching,^25^ and the peak at 3306 cm^−1^ corresponded to H–H bonds and the phenolic functional group. Further, the peaks at 2357 and 923 cm^−1^ were associated with atmospheric CO_2_ (ref. 26) and the bending vibration of the RCO–OH carboxylic functional group. The peaks at 685, 607 and 484 cm^−1^ were ascribed to the stretching vibration of the C–OH bond.^27,28^ Thus, the FT-IR results suggested the formation of the pm-CuO NPs. Further, in the zeta-potential analysis of pm-CuO NPs, a negative potential of −21.5 mV was displayed (see ESI Fig. S3 in Section 2†); from this, it can be stated that these biogenic nanoparticles are highly bioactive and will remain stable in the colloidal suspension.

The X-ray diffraction (XRD) pattern revealed the orientation and crystalline nature of the pm-CuO NPs (Fig. 2D). The peaks standing state 2*θ* values were 34.10°, 35.63°, 38.83°, 49.00°, 53.50°, 58.22°, 61.78°, 66.16°, 68.04°, 72.40°, and 75.14°, representing the (002), (111), (111), (202), (020), (202), (113), (311), (220), (311), and (222) planes, respectively, which were attributed to the monoclinic phase of pm-CuO NPs with a crystalline structure, as all the corresponding peaks agreed with the Joint Committee's database on Powder Diffraction Standards (JCPDS card no. 89-2529). In this obtained result, no additional peaks were observed that would demonstrate any impure phase. The standard lattice parameters obtained from the JCPDS card were *a* = 4.6832 Å, *b* = 3.4288 Å, *c* = 5.1297 Å, corresponding to the monoclinic structure of these pm-CuO NPs. We further calculated the average crystallite size by using the Scherrer formula (eqn (1)) from the obtained crystallographic planes, and the calculated average crystalline size from eqn (1) was 11 nm. In eqn (1)*D* corresponds to the crystallite size, the X-ray wavelength *λ* = 1.5406 Å, *β* is the full width at half maximum (FWHM) of the more intense peak, and the Bragg diffraction angle is represented by *θ*.

1 *D* = 0.9*λ*/(*β* cos *θ*)

**Fig. 3:**
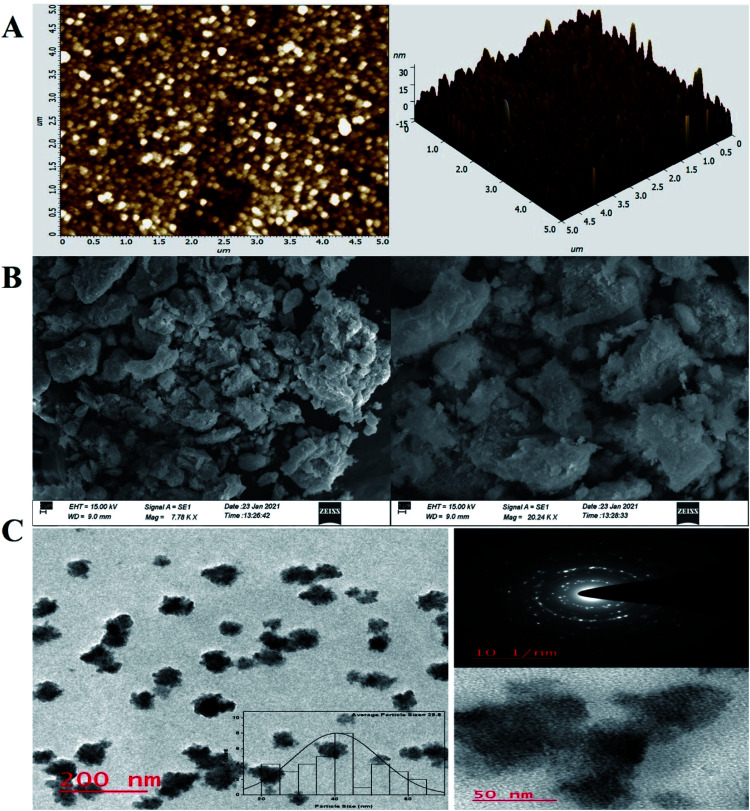
AFM, SEM and TEM analyses of the pm-CuO NPs, (A) AFM data of the pm-CuO/ITO electrode 2D (left), and 3D (right), (B) pm-CuO NPs micrography by SEM, and (C) TEM analysis of the pm-CuO NPs; inset, histograms of the average particle size (left); zoom portion of left TEM image demonstrating NPs, SAED analysis, and HR-TEM of the pm-CuO NPs (right).

**Fig. 4:**
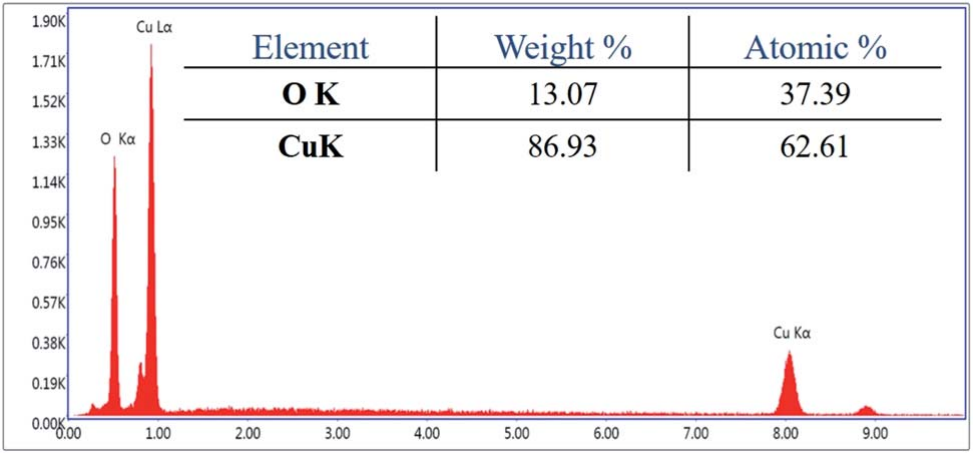
EDX analysis of pm-CuO NPs.

For investigation of the pm-CuO/ITO electrode's surface properties, atomic force microscopy (AFM) studies were carried out, and the results shown in Fig. 3A. The pm-CuO/ITO electrode morphology demonstrated a uniform distribution of pm-CuO NPs on the ITO surface, and from the roughness calculation of the CuO electrode surface, the root mean square roughness (RMS-*R*_q_), roughness average (*R*_a_), and the max peak height profile were 5.187 nm, 4.041 nm, and 32.807 nm, respectively. Further, it was also observed that the pm-CuO NPs on the surface of ITO were distributed uniformly, and the distributed particles were spherical, and the B–CuO/ITO electrodes three-dimensional (3D) surface morphology also demonstrated the homogeneous distribution of spherical structural elements. Further, the surface topology of the pm-CuO NPs powder was validated by scanning electron microscopy (SEM; Fig. 3B), and it demonstrated again that the pm-CuO NPs were uniformly distributed, all in relation to the obtained AFM data; however, agglomeration was also observed to some extent. Apart from these studies, transmission electron microscopy (TEM) was also utilized to analyze the pm-CuO NPs particles' size and shape, as demonstrated in Fig. 3C. The TEM results revealed that most of the pm-CuO NPs were spherical, and the quantitative analysis performed by using the TEM image revealed the average particle size of the pm-CuO NPs to be around 39.8 nm, as determined by a histogram fitted by the Lorentzian function. Thus, the average particle size and crystalline particle size as calculated by XRD were related as the crystalline size of a particle can be ±20 nm less than the average particle size of NPs; in addition to this, the SAED (selected area electron diffraction) image (Fig. 3C) obtained from TEM was also in agreement with the *d*-spacing values of the JCPDS data and corresponded to the highly intense peak in the XRD; from this, it can again be concluded that the SAED image is in complete agreement with the 2*θ* peaks obtained in the XRD analysis and that the SAED image also demonstrated the highly polycrystalline nature of the synthesized pm-CuO NPs. Finally, it was very important to confirm the synthesized products' elemental compositions, which were examined through EDX, and the obtained result is shown in Fig. 4, whereby it was confirmed from this that the synthesized NPs were CuO as the EDX demonstrated the presence of only two elements, namely Cu and O, with atomic% values of 62.61 and 37.39, respectively.

### 2.2. Antibacterial studies

**Fig. 5:**
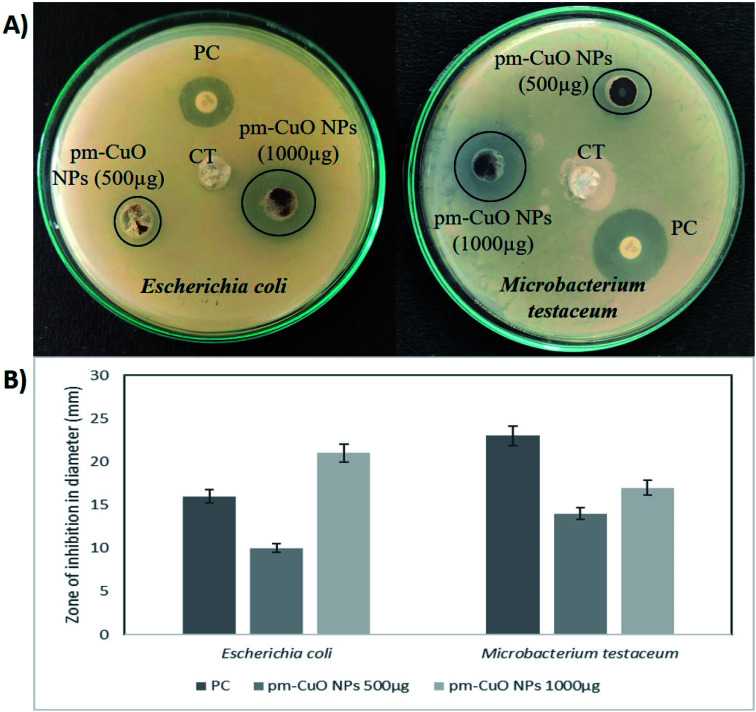
Antibacterial activity of the pm-CuO nanoparticles: (A) well-diffusion antibacterial assay of pm-CuO NPs for Gram-negative (*Escherichia coli*) and Gram-positive (*Microbacterium testaceum*) bacterial strains. CT, Control (plant extract – *Annona squamosa*); PC, Positive control (*Ampicillin*). (B) Zone of inhibition of PC and different concentrations of pm-CuO NPs (500 and 1000 μg) on *E. coli* and *M. testaceum*.

In nature, crop plants are confronted by various bacterial pathogens leading to severe crop losses, resulting in global socio-economic disturbances. Therefore, controlling plant diseases using antibacterial agents without affecting the environment is a pressing need in agriculture. In this work, biologically synthesized pm-CuO NPs potentialities were first examined on two bacteria, *Escherichia coli* (Gram-negative) and *Microbacterium testaceum* (Gram-positive), by a well-diffusion method. The antibacterial well-diffusion assay was performed in triplicate, and the pm-CuO NPs at both the tested concentrations (500 and 1000 μg) exhibited a great zone of inhibition (ZOI) against both *E. coli* and *M. testaceum*. At a concentration of 1000 μg of pm-CuO NPs, the ZOIs against both *E. coli* and *M. testaceum* were 21 and 17 mm in diameter, respectively (Fig. 5A and B). The relatively higher ZOI against *E. coli* suggested that the biological NPs could be a more effective antibacterial agent against Gram-negative bacteria. Various mechanisms can be attributed to the antibacterial effect of pm-CuO NPs against both Gram-positive and Gram-negative bacteria. CuO NPs were previously shown to interact with the plasma membrane of bacteria, leading to holes in the membrane and the lysis of bacterial cell.^29^ The released Cu^2+^ ions were also found to interact with negatively charged components of the cell wall and membrane, leading to denaturation and alteration of the membrane proteins.^30^ Copper ions inside the bacterial cell generate ROS, leading to alterations in cellular signalling and interfering with the nucleic acid, resulting in an altered helical structure.^31^

**Fig. 6:**
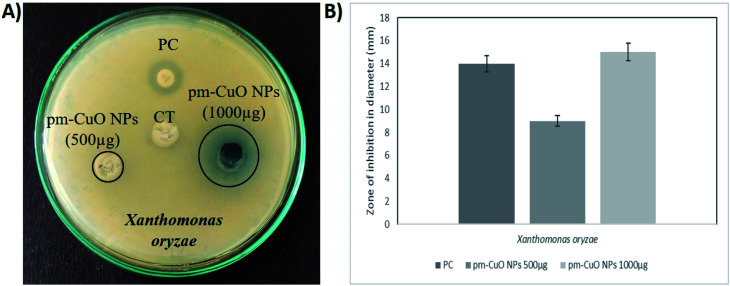
Antibacterial activity of the pm-CuO nanoparticles: (A) well-diffusion antibacterial assay of pm-CuO NPs for plant pathogenic Gram-negative bacteria (*Xanthomonas oryzae*). CT, Control (plant extract – *Annona squamosa*); PC, Positive control (*Ampicillin*). (B) Zone of inhibition of PC and different concentrations of pm-CuO NPs (500 and 1000 μg) on *Xanthomonas oryzae*.

Herein this study, we hypothesized that the pm-CuO NPs have a great potential for agricultural applications as an antibacterial agent to fight against plant pathogenic bacteria. As in the preliminary studies, the Gram-negative bacteria *E. coli* showed higher sensitivity towards the pm-CuO NPs. Consequently, we further selected a Gram-negative plant pathogenic bacterium, *Xanthomonas oryzae*. The major host of this bacterium is rice (*Oryza sativa*), in which it causes bacterial blight and affects ∼75% of the rice crop annually. The major regions in which the rice crop is highly affected by this pathogen are Asia, the Caribbean, Latin America and Africa's western coast.^32^ The antibacterial well-diffusion assay against *X. oryzae* showed sensitivity towards both 500 and 1000 μg pm-CuO NPs by exhibiting 9 and 15 mm ZOIs, respectively (Fig. 6A and B). Hence, from the antibacterial study, it is evident that the biogenic pm-CuO NPs have the potential to be utilized as an agent for combating the bacterial blight disease caused by *X. oryzae*, but this is still subject to further studies related to the nanotoxicological aspect before the on-field utilization of this material as an antibacterial agent is possible.

### 2.3. Electrochemical studies

#### 2.3.1. CV and DPV

**Fig. 7:**
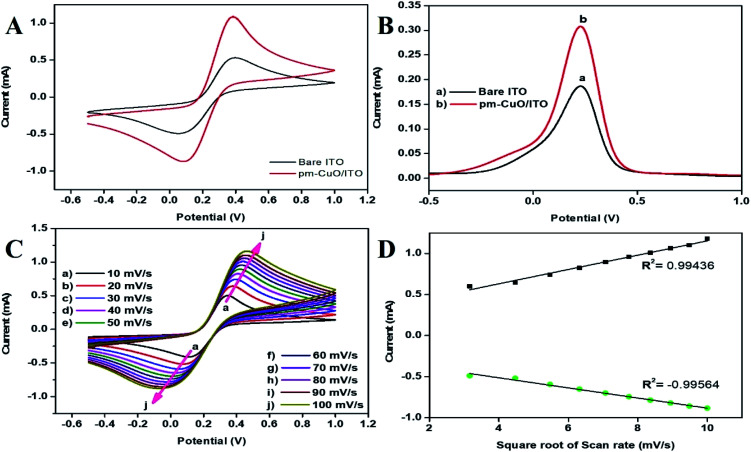
(A) CV and (B) DPV of the bare ITO (black curve), and pm-CuO/ITO (red curve) electrodes, (C) CV curve of pm-CuO/ITO at varying scan rates (10–100 mV s^−1^, pH 5.7) at 27 °C. (D) Increasing current *vs.* difference in potential as a function of the square root of the scan rate (10–100 mV s^−1^).

The redox behaviour of pm-CuO/ITO electrodes prepared by electrophoretic deposition (see ESI Section 1.4†) were investigated in 50 mM PBS (0.9% NaCl) solution of pH 5.7 containing 5 mM [Fe(CN)_6_]^3−/4−^at a 50 mV s^−1^ scan rate, in comparison with the bare ITO electrode (Fig. 7A). The cyclic voltammetry (CV) results of the bare ITO (black curve) exhibited a redox couple of peaks, which corresponded to the mediator [Fe(CN)_6_]^3−/4−^, which demonstrated the electrochemical characteristics, while the bare ITO had an oxidation/anodic current peak (*I*_pa_) at 0.53 mA. For the electrodeposited pm-CuO/ITO electrode, the *I*_pa_ value was highly elevated and gave a current peak at 1.09 mA (red curve), which was because the pm-CuO NPs provided relatively better conductivity owing to their large surface area, which further indicated the superior electrocatalytic behaviour compared to ITO as an electrode. The differential pulse voltammetric (DPV) studies result were also related to the corresponding CV measurements, as a higher current was exhibited on the pm-CuO NPs fabricated electrode (Fig. 7B). Further, the CV analyses of the bare ITO and pm-CuO/ITO electrodes (ESI Fig. S4†) in 50 mM PBS (0.9% NaCl) solution of pH 5.7 without the mediator [Fe(CN)_6_]^3−/4−^ were performed, and demonstrated no sharp oxidation or reduction behaviour, which highlighted that the superior electrocatalytic behaviour was the result of the mediator [Fe(CN)_6_]^3−/4−^, which offered the pm-CuO/ITO electrode a redox potential.

#### 2.3.2. Scan rate effect

For investigating the interfacial kinetics of the pm-CuO/ITO electrode as a function of varying the scan rate from 10 mV s^−1^ to 100 mV s^−1^, CV studies were conducted (Fig. 7C). From this study, it was obvious that the magnitude of the anodic/oxidation (*I*_pa_) and cathodic/reduction (*I*_pc_) peaks current increased linearly with respect to the scan rate (Fig. 7D). Moreover, with the increase in the scan rate, the current at the oxidation peak (*I*_pa_) shifted towards a more positive potential value, whereas the current at the reduction peak (*I*_pc_) shifted towards a more negative potential, which in turn, suggests that the oxidation–reduction (redox) behaviour was a quasi-reversible process.

#### 2.3.3. Kinetic studies

The charge-transfer rate constant (*K*_s_) value changes obtained by the surface modification of ITO by the pm-CuO NPs were calculated by eqn (2),^33^ where *F* is the Faraday constant (96 485 C mol^−1^); *R* is the gas constant, which is 8.314 J mol^−1^ K^−1^; *m* is a separation of the peak-to-peak (V); *n* corresponds to the number of electrons transferred, *i.e.*, 2 in the H_2_O_2_; *ν* (50 mV s^−1^) stands for the scan rate; and *T* is 27 °C (room temperature). By employing all these values, *K*_s_ was determined for THE pm-CuO/ITO electrode to be 1.508666 s^−1^, which was attributed to the increased transfer of electrons owing to the higher catalytic activity of the pm-CuO NPs. The shift in potential (Δ*E*_p_ = *E*_pa_(anodic peak) − *E*_pc_(cathodic peak)) exhibited a linear relationship with respect to the scan rate (Fig. 7D). From this result, it was evident that the electrochemical reaction of H_2_O_2_ with the pm-CuO NPs was a diffusion-controlled process. Further, the *D* (diffusion coefficient) for the diffusion of [Fe(CN)_6_]^3−/4−^ from the electrolyte solution to the pm-CuO/ITO electrode surface was investigated by the Randles–Sevcik equation, eqn (3),^33^ in which *I*_p_ corresponded to the electrode peak current, *i.e.*, *I*_pa_ and *I*_pc_; *n* is the electron number (2), *A* is the electrode surface area (0.25 cm^2^), and *C* is the concentration at the surface in mol cm^−3^ (5 mM). Putting the values in eqn (3), the obtained *D* value of the pm-CuO/ITO electrode was 3.241 × 10^2^ cm^2^ s^−1^. The surface concentration of the electrode was projected by the Brown–Anson model (eqn (4)), where *n* is the electron number transferred, *i.e.*, 2, *γ* corresponds to the electrode's surface concentration (mol cm^−2^), and *T* is room temperature. Thus, the pm-CuO/ITO's surface concentration can be calculated by putting all the above values in eqn (4) and it was found to be 9.34 × 10^−8^ mol cm^−2^.

2 *K*_s_ = *mnFν*/*RT*

3 *I*_p_ = (2.69 × 10^5^)*n*^3/2^*AD*^1/2^*Cν*^1/2^

4 *I*_p_ = *n*^2^*F*^2^*γAV*/4*RT*

#### 2.3.4. Optimization of the pH

**Fig. 8:**
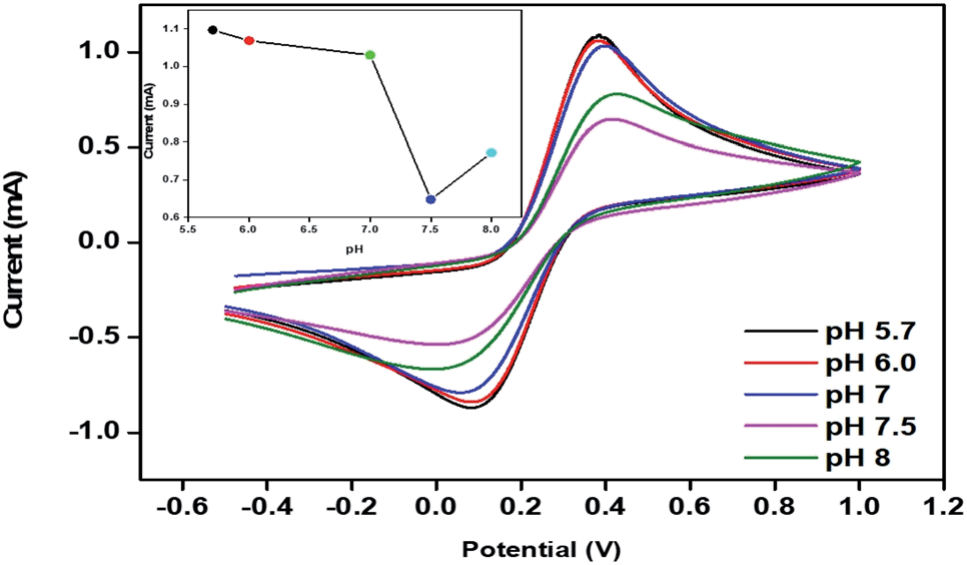
CV of the pm-CuO/ITO electrode with varying the pH (5.7 to 8) of 50 mM PBS (0.9% NaCl) containing 5 mM [Fe(CN)_6_]^3−/4−^; inset shows the current response of the pm-CuO/ITO electrode as a function of the pH.

The electrochemical behaviour of pm-CuO/ITO was optimized for different pH values (5.7 to 8.0) by CV studies in 50 mM PBS (0.9% NaCl) containing 5 mM Fe[(CN)_6_]^3−/4−^ at a scan rate of 50 mV s^−1^ (Fig. 8). It could be observed that the oxidation/anodic peak current decreased from pH 5.7 to 7.5, and at pH 8, the *E*_pa_ value again increased slightly. The maximum current at *E*_pa_ was observed at pH 5.7 due to the interaction between the pm-CuO/ITO with the [Fe(CN)_6_]^3−/4−^ compared to the current at the *E*_pa_ of pm-CuO/ITO at other pH conditions. Hence, pH 5.7 was determined to be a suitable pH for performing the CV measurements after optimization.

#### 2.3.5. Electro-oxidation studies of H_2_O_2_

**Fig. 9:**
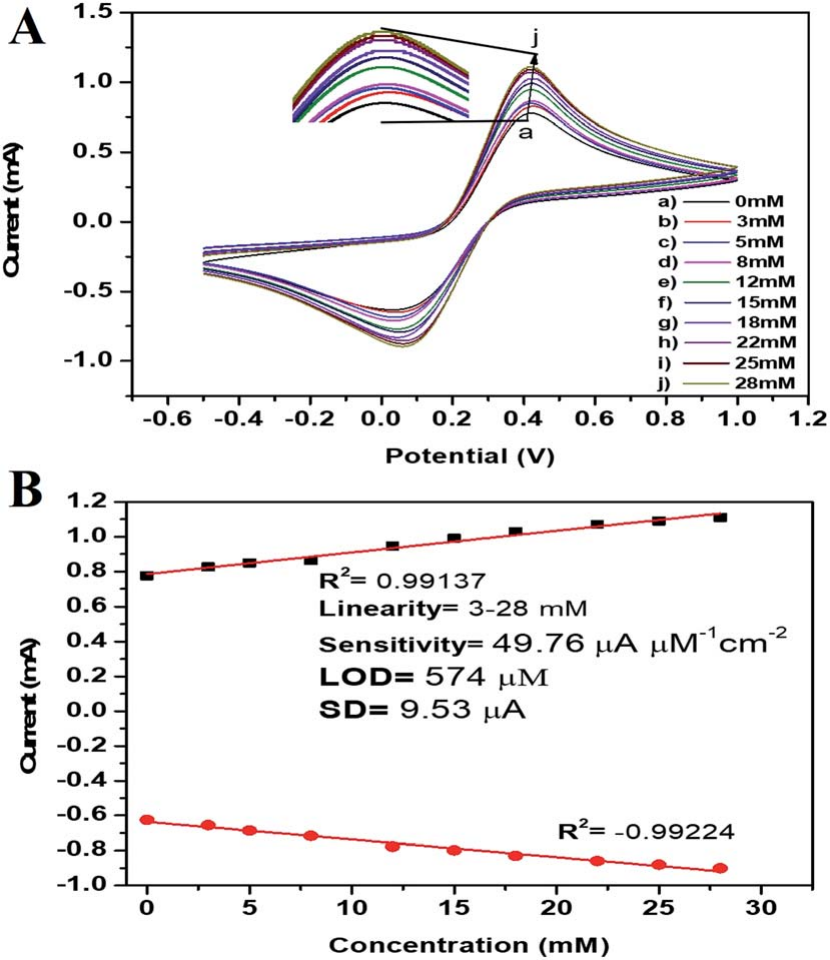
(A) Electrochemical sensing of varying concentrations of H_2_O_2_ (3–28 mM) by the pm-CuO/ITO electrode in 50 mM PBS (pH 5.7, 0.9% NaCl) containing 5 mM [Fe(CN)_6_]^3−/4−^ at a scan rate of 50 mV s^−1^. The inset shows the zoomed view of the current response obtained by the pm-CuO/ITO electrode. (B) Calibration curve of the pm-CuO/ITO electrode with the variation of the current as a function of the H_2_O_2_ concentration (3–28 mM) in 50 mM PBS (pH 5.7, 0.9% NaCl) containing 5 mM [Fe(CN)_6_]^3−/4−^ at a scan rate of 50 mV s^−1^.

**Fig. 10:**
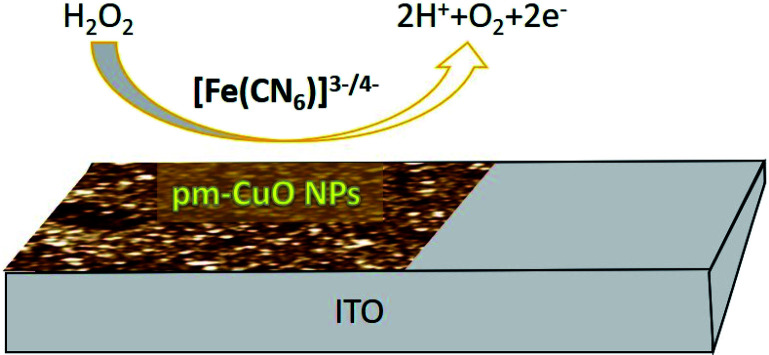
Diagrammatic illustration of the reduction reaction mechanism of H_2_O_2_ at the pm-CuO/ITO electrode.

In this work here we hypothesized that the biogenic pm-CuO NPs have potential applications in the agricultural domain, and to prove this hypothesis, we demonstrated the electrochemical sensing response to H_2_O_2_, as it is very toxic to plants when produced in a higher concentration during plant stress conditions and it results in oxidative damage to plants and if not controlled it will lead to plant death. Thus, there is an urgent need to develop a highly effective sensor for determining the plant's chemicals during stress conditions, which can help treat plants with supplements to overcome the stress. For achieving this objective, the CV response of the pm-CuO/ITO electrode was analyzed with varying the H_2_O_2_ concentration in 50 mM PBS (pH = 5.7, 0.9% NaCl) containing [Fe(CN)_6_]^3−/4−^ at a 50 mV s^−1^ scan rate. Fig. 9A shows the electrochemical sensing according to the CV results of the pm-CuO/ITO electrode as a function of the varying H_2_O_2_ concentration (3 to 28 mM). From the CV studies, it is evident that with the increasing concentration of H_2_O_2_, the anodic and cathodic peak current increased and decreased, respectively, at linear intervals, which suggested that the pm-CuO NPs were very sensitive to H_2_O_2_. The overall reaction for reducing H_2_O_2_ by the pm-CuO/ITO electrode can be written as demonstrated in Fig. 10 and eqn (5).

5 H_2_O_2_ → 2H^+^ + O_2_ + 2e^−^

The peak current obtained at the anodic/oxidation and cathodic/reduction peak showed a linear relationship with the concentration range between 3–28 mM (Fig. 9B). The linear calibration coefficient (*R*^2^) obtained was 0.99137, demonstrating a very strong relationship between the H_2_O_2_ concentration and the obtained anodic/oxidation current. The fabricated pm-CuO/ITO electrode showed a very low detection limit (DL)/limit of detection (LOD) of 575 μM and demonstrated linearity from 3–28 mM. Moreover, the as-fabricated electrode had a sensitivity of 49.76 μA μM^−1^ cm^−2^, which indicated it was highly sensitive. The LOD was calculated by 3 × SD/sensitivity, where SD stands for the standard deviation of the calibration plot slope and background current. From the pm-CuO/ITO electrodes electro-oxidation activity results when exposed to various H_2_O_2_ concentrations, it could be concluded that this electrode could be used as a prominent sensor for the quantitative determination of H_2_O_2_ in any agricultural samples as well as any biological samples too. Further, the sensing response of the as-fabricated electrode towards H_2_O_2_ of different concentrations (3, 12, 22 and 28 mM) was also checked in 50 mM PBS (0.9% NaCl) solution at pH 5.7 without the mediator [Fe(CN)_6_]^3−/4−^ (ESI Fig. S5†), and demonstrated no response towards H_2_O_2_, which might be due to the absence of the redox-centre in the as-fabricated electrode. Therefore, it was evident that the fabricated electrode was not suitable for sensing H_2_O_2_ without the mediator [Fe(CN)_6_]^3−/4−^. For the response time determination of the pm-CuO/ITO electrode, the electrochemical response was measured from 5 to 60 seconds, and initially the current magnitude increased and reached a maximum value at 40 seconds, and after this, the current started decreasing, which demonstrated that 40 seconds was the response time of the pm-CuO/ITO electrode for the proper sensing of H_2_O_2_. Moreover, interference studies (ESI Fig. S6A†) were also performed in the presence of ascorbic acid (12 mM), urea (12 mM), and dextrose (12 mM), which demonstrated no significant current change. Furthermore, reproducibility/reusability studies were also performed (ESI Fig. S6B†), which demonstrated that after 25 scans, the pm-CuO/ITO electrode lost only 0.03% of the current, which demonstrated that this label-free electrode was highly reusable/reproducible. Stability studies (ESI Fig. S6C†) were also performed for 51 days at an interval of 5 days, and the pm-CuO/ITO electrode retained its activity up to 51 days, as on the 51st day it had only lost 0.09% current, which justifies that the as-fabricated electrode was highly stable for up to 51 days at 4 °C. Further, Table 1 compares this work for the electrochemical sensing of H_2_O_2_ with pre-existing work of this type but utilizing different materials as an electrode.^34–36^ The sensitivity obtained in this work was very high when compared to other work, but the response time in the other works was much less, albeit our as-fabricated electrode showed a delayed response towards H_2_O_2_, which may be due to the slow oxidation and reduction potential of the pm-CuO NPs when compared with Pd and other composite materials based on Ce, polyaniline, Na, *etc.*

**Table 1:** Demonstrates the electro-analytical parameters of the previous work and compares them with this work

Methods	Electrode	pH, medium	Sensitivity (μA μM^−1^ cm^−2^)	Liner range	Response time (s)	Ref.
CV	Pd	6.8, PBS	0.368	53 to 4400 μM	—	34
CV	HRP/nano CeO_2_ bioelectrode	7.0, PBS	8.44	1.0–170 μM	3	35
CV	(Na, O-*g*-C_3_N_4_/GCE)	—, PBS	3.41	1–50 μM	—	36
CV	B–CuO/ITO	5.7, PBS	49.76	3–28 mM	40	Present work

## 3. Conclusion and prospects

In this work, biogenically fabricated CuO NPs were characterized for their optical, structural and morphological properties and then further investigated for their applications in the agricultural domain by checking the pm-CuO NPs utility as an antibacterial agent for combating plant pathogenic bacterium and the utilization of pm-CuO NPs to fabricate a label-free sensor for the quantitative determination of H_2_O_2_ in plants for taking proper measures to help plants combat stress conditions. Further, we performed initial work to prove the utility of pm-CuO NPs as an antibacterial agent, and work also proves that the pm-CuO NPs could be very much beneficial for combating the bacterial blight disease in the rice crop. However, before utilization of this material on-field for combating bacterial blight disease, it has to undergo nanotoxicological studies, and our next objective is to achieve this. Moreover, herein we also report the role of pm-CuO NPs for the electro-oxidation response towards H_2_O_2_, which demonstrated a very good response time, high sensitivity and low LOD of 40 seconds, 49.76 μA μM^−1^ cm^−2^, and 574 μM, respectively. The sensing of H_2_O_2_ was highly linear in the linear range from 3–28 mM. Thus, this study's findings propose pm-CuO NPs as a promising agent to be utilized in agriculture as an antibacterial agent for combating bacterial blight disease. It can also be proposed as a capable platform for the label-free, simple and rapid quantitative determination of H_2_O_2_ in the agricultural samples of plants/crops, for the proper monitoring of stress conditions, as per during normal conditions in the plant, when H_2_O_2_ is produced but in a much lower concentration, which does not harm them. However, during stress, H_2_O_2_ is produced in a higher concentration, which causes oxidative damage to the cell and even death. To avoid this, the CuO-based H_2_O_2_ sensors can be a prominent tool for the determination of H_2_O_2_ levels in plants. If the sensor determines the increased H_2_O_2_ levels, then necessary measures can be taken by supplementing the plants with vitamin C or another supplements to avoid plant/crop death from oxidative damage. The work reported in this is preliminary information, and we are also working on models for the commercial availability of our biogenic pm-CuO NPs-based sensors for on-field detection of H_2_O_2_, but currently, we are working towards further optimization of this sensor for making it cost effective, robust, and more accurate, and for this, we are working on the fabrication of a paper-based electrode with the same material to achieve a similar sensitivity and effectiveness for making this technology cost effective.

## Author contributions

PS contributed to the data curation, investigation, resources, validation and writing original draft. KRBS contributed to the conceptualization, data curation, investigation, resources, validation, writing an original draft and reviewing & editing the manuscript draft. JS contributed by data curation, resources, supervision, visualization, writing original draft, and editing the manuscript draft. SND contributed by conceptualization, data curation, supervision, validation, and reviewing & editing the manuscript draft. RPS contributed by conceptualization, data curation, validation, project administration, supervision, writing original draft, and reviewing & editing the manuscript draft.

## Conflicts of interest

The author's declare that they have no conflicts of interest for this work.

## Supplementary Material

RA-011-D1RA02382A-s001
