# Supplementary material for: Tunable electrochemistry and efficient antibacterial activity of plant-mediated copper oxide nanoparticles synthesized by Annona squamosa seed extract for agricultural utility
Source: RSC Adv. 2021 May 19;11(29):18050–60. doi: 10.1039/d1ra02382a (PMC9033215; doi:10.1039/d1ra02382a)
Supplement: RA-011-D1RA02382A-s001 [file RA-011-D1RA02382A-s001.pdf]

Electronic Supplementary Material (ESI) for RSC Advances.
This journal is © The Royal Society of Chemistry 2021

Tunable electrochemistry and efficient Antibacterial activity of plant mediated-Copper Oxide Nanoparticles Synthesized by Annona squamosa Seed Extract for agricultural utility

Pooja Singh[1], Kshitij RB Singh[2], Jay Singh[3], Subha Narayan Das[4], Ravindra Pratap Singh[*, 1]

[1]Department of Biotechnology, Indira Gandhi National Tribal University, Amarkantak, M.P. (484886), India.

[2]Department of Chemistry, Govt. V. Y. T. PG. Autonomous College, Durg, Chhattisgarh, India

[3]Department of Chemistry, Institute of Science, Banaras Hindu University, Varanasi, U.P. (221005), India

[4]Department of Botany, Indira Gandhi National Tribal University, Amarkantak, M.P. (484886), India.

[*]**Corresponding Author:**

Dr. Ravindra Pratap Singh,

Assistant Professor,

Department of Biotechnology,

Faculty of Science,

Indira Gandhi National Tribal University,

Amarkantak, Madhya Pradesh (484886), India.

Email Id- rpsnpl69@gmail.com, ravindra.singh@igntu.ac.in

Mobile No.- +91-91-0934-6565.

Table of Content

1. Experimental

1.1. Material

1.2. Preparation of AS plant extract

1.3. Biological synthesis of pm-CuO NPs

1.4. Electrophoretic deposition of pm-CuO/ITO film

1.5. Characterization

2. Zeta potential data

3. CV Studies without mediator $[Fe(CN)_6]^{3-/4-}$

4. Electrochemical Sensing of H_2O_2 without mediator $[Fe(CN)_6]^{3-/4-}$

5. Interference, Reproducibility, and Stability Studies

1. Experimental

1.1. Material

Copper sulphate pentahydrate ($CuSO_4.5H_2O$; MW: 249.68 g/mol; CAS Number: 7758-99-8), Hydrogen peroxide (H_2O_2; MW:34 g/mol; Product Cat no.: 1.93007.0521), Potassium hexacyano ferrate(II) trihydrate ($C_6FeK_4N_6.3H_2O$; MW: 422.39 g/mol; Product cat no.: 1.93686.0521), Potassium hexacyano ferrate (III) ($C_6FeK_3N_6$; MW: 329.25 g/mol; Product cat no.: 1.93667.0521), and Sodium chloride (NaCl; MW: 58.44 g/mol; Product Cat No.: S9888), Whatman filter paper Grade-1 were obtained from Sigma-Aldrich. Sodium hydroxide (NaOH; MW: 40.00 g/mol; CAS No.: 1310-73-2) and Liquor Ammonia (NH_3; MW: 17.03 g/mol; CAS No.: Q16225) obtained from Qualigens, Thermo Fisher Scientific. Luria Bertani Agar, Miller (GM1151), Ampicillin (SD002), Disodium Phosphate ($Na_2HPO_4.H_2O$; MW: 177.99; CAS No.: 10028-24-7), and Monosodium Phosphate (NaH_2PO_4; MW: 119.98; CAS No.: 7558-80-7) were procured from Himedia. All the chemicals used for experimenting were of analytical grade, and it was used as received without further purification. The ITO sheet (surface resistivity 30-60 Ω/sq) was procured from Sigma-Aldrich for electrophoretic deposition of Biogenic copper oxide nanoclusters (B-CuO NCs). The pure culture of Gram-positive (*Microbacterium testaceum*) and gram-negative (*Escherichia coli and Xanthomonas oryzae*) bacteria were laboratory obtained. *Annona squamosa (AS)* fruit was procured from the local market Amarkantak, M.P., India. Milli-Q water (18Ω resistivity), Millipore, USA was used to perform all the experimental works.

1.2. Preparation of AS plant extract

From the collected AS fruit, the seeds were isolated and washed with Milli-Q water 2-3 times to remove the fruit's pulp and then dried in sunlight for two weeks; after drying, the seeds were again washed and kept at ambient temperature for removal of water content. Further, the dried seeds were crushed using mortar and pestle, followed by a mixer to grind the crushed seeds

into a fine powder. The prepared AS powder was exactly weighed 2 gm and dispensed into an ultraclean beaker containing 50 ml of Milli-Q water, and further, more water was added to make up 100ml. After adding it, the solution was boiled at 100^oC for 30 minutes. After 30 minutes of boiling, the extract solution was kept at room temperature for lowering of temperature. Finally, the cooled solution was centrifuged at 10000 rpm for 10 minutes; the pellet was discarded, and the supernatant was retained; further, the supernatant was filtered through the Whatman filter paper 1; the obtained filtrate solution was stored at 4 ^oC for further experiments.

1.3. Biological synthesis of pm-CuO NPs

For the synthesis of pm-CuO NPs initially, 5M of $CuSO_4.5H_2O$ salt stock solution was prepared in Milli-Q water, and the pH of the salt solution was found to be ~3. Further, for preparing pm-CuO NPs, the room temperature was preferred after completed optimization of different pH, salt concentration and plant extract concentration; after complete optimization of pm-CuO NPs synthesis on the parameter mentioned above, the highly yielded NPs in the best salt concentration with respect to pH and plant extract concentration was selected for final synthesis which is pH 14 in equivolume of 1M $CuSo_4.5H_2O$ salt and plant extract. Thus, for the final biological synthesis of pm-CuO NPs, 1M 100ml $CuSO_4.5H_2O$ solution was kept on a magnetic stirrer at 400rpm, then 100ml of plant extract was added slowly to it and kept on constant stirring for 1 hour at room temperature. Further, 5M NaOH was added in a dropwise manner to raise the reaction mixture's pH to 14pH, and as a result of pH increase, the color of the reaction mixture changed drastically from light blue to dark bluish, and a high amount of precipitate was observed. After maintaining the pH, the reaction mixture was again kept at room temperature with continuous stirring at 400rpm for 8 hours, and after this, the color changes from dark bluish to brown color, which indicates the formation of pm-CuO NPs. After this, the synthesized NPs were washed 3-5 times by Milli-Q water followed by centrifugation

at 10000rpm for 10 minutes. Further, the washed dark brown pellets were air-dried by a hot air oven at 70 ^{O}C for 12hours. After air-drying, the synthesized pm-CuO NPs were stored in an airtight container for further characterization and applications.

1.4. Electrophoretic deposition of pm-CuO NPs on ITO

In this work, we will utilize the electrophoretic deposition (EPD) method to make a thin film of prepared pm-CuO NPs as illustrated in **supp. figure 1**. For performing EPD, ITO glass substrate was cut into 1x2cm (length x breadth) then it was hydrolyzed by 5:2:2 (double distilled water: hydrogen peroxide: Ammonia) solution by completely immersing in this solution and incubated for 1 hour at 60 ^{0}C. After 1-hour incubation, it was kept at room temperature for 15 minutes to lower the temperature. After the decrease in temperature, remove the ITO from the solution and water wash 3-4 times and let it dry and place it in a desiccator to avoid dust and moisture before use. Further, after optimization of all the EPD parameters, 1 mg of prepared pm-CuO NPs was added to 20ml of 20% ethanol-water mixture (20% ethanol and 80% Milli-Q water), and then it was kept for 30 minutes of ultrasonication for forming colloidal dispersion. Further, the prepared pm-CuO/ITO electrode was used for performing electro-oxidation studies of H_2O_2 by utilizing the three-electrode system as depicted in **supp. figure 2**.

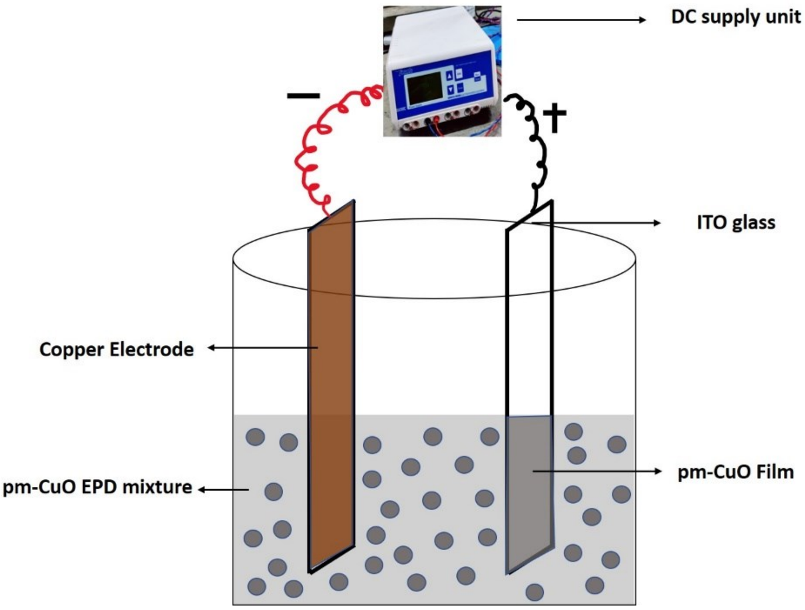

Supp. Figure 1. Diagrammatic illustration of EPD of pm-CuO NPs onto the ITO surface.

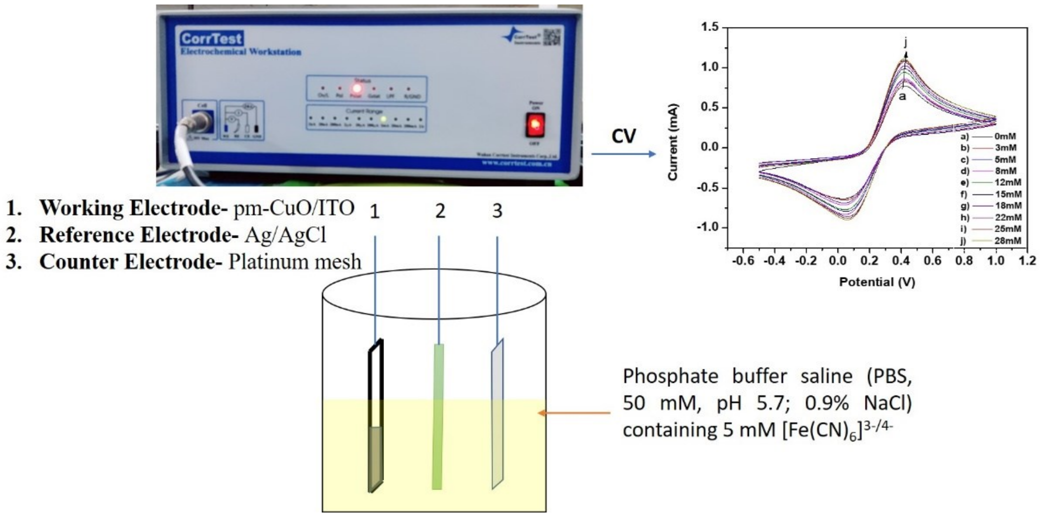

Supp. Figure 2. Complete overview of electrochemical sensing of H_2O_2 on three electrode system by utilizing pm-CuO/ITO electrode.

1.5. Characterization

UV-vis absorption spectroscopy was performed by using UV-1800 Shimadzu, Japan, in the wavelength range of 200-800 nm to investigate NPs optical properties. The vibration spectrum of the synthesized biogenic pm-CuO NPs was determined by FTIR spectrophotometer (Nicolet

iS5, Thermo Fisher Scientific) in the spectral range 400-4000cm^{-1}. XRD techniques examined the structure and crystalline size of pm-CuO NPs by using an X-ray diffractometer (D8 Advance, Bruker) with a Cu-Kα (λ = 1.5406 Å) in the 2θ angles ranging from 10° to 80°. Zetasizer (Litesizer 500, Anton Paar) was used to determine the zeta potential of the synthesized material. SEM and energy dispersive X-ray analysis (EDX) were performed to determine surface morphology and element confirmation, respectively, of synthesized NPs by using EVO 18, Zeiss. TEM analysis was performed for pm-CuO NPs elemental imaging by utilizing FEI Tecnai G2 F20-Twin, Swiss Republic, at an operating voltage of 300 kV. AFM (NEXT, NT-MDT) was used to determine the fabricated pm-CuO/ITO electrodes surface morphology. Further, the electro-oxidation study of H_2O_2 by the pm-CuO/ITO electrode was performed by CorrTest, electrochemical workstation, Wuhan Corrtest Instruments, China, using three electrodes system as demonstrated in the **supp. figure 2**.

2. Zeta potential data

Supp. Figure 3 demonstrates the mean zeta potential value of pm-CuO NPs.

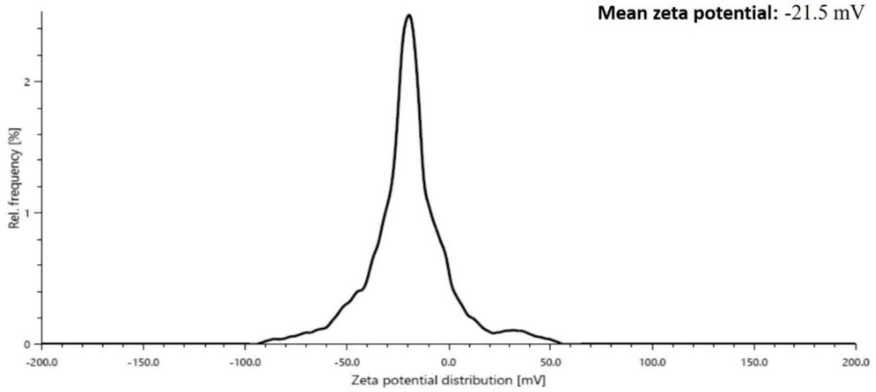

Supp. Figure 3. Mean Zeta potential of pm-CuO NPs.

3. CV Studies without mediator $[Fe(CN)_6]^{3-/4-}$

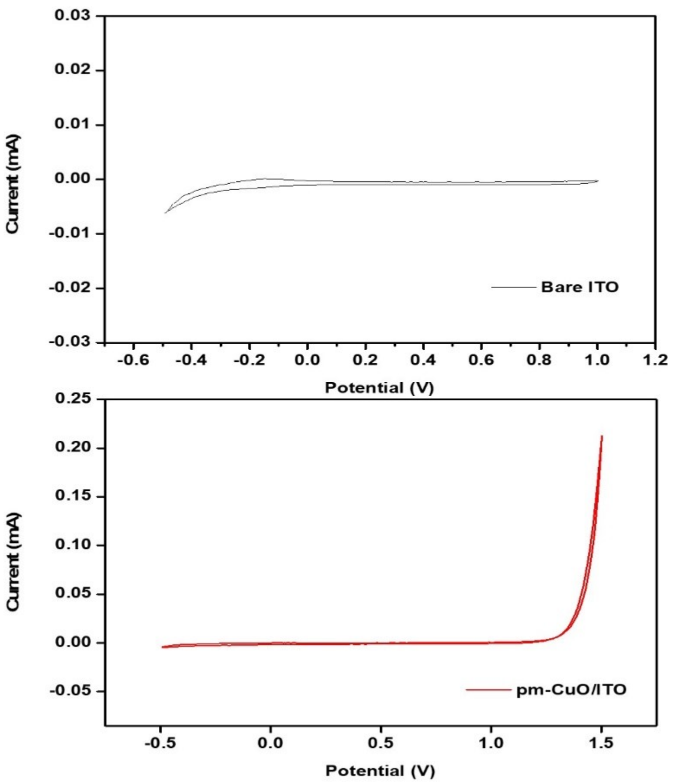

Supp. Figure 4. CV of Bare ITO (black curve) and pm-CuO/ITO (red curve) in 50mM PBS (0.9% NaCl) solution of pH 5.7.

4. Electrochemical Sensing of H_2O_2 without mediator $[Fe(CN)_6]^{3-/4-}$

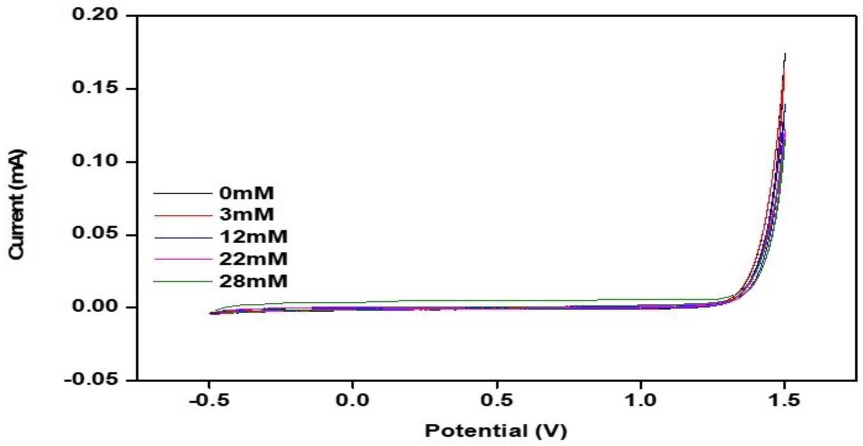

Supp. Figure 5. Electrochemical sensing of H_2O_2 varying concentrations (3, 12, 22, and 28mM) by pm-CuO/ITO electrode in 50mM PBS (pH 5.7, 0.9% NaCl) at scan rate of 50mV/s.

5. Interference, Reproducibility, and Stability Studies

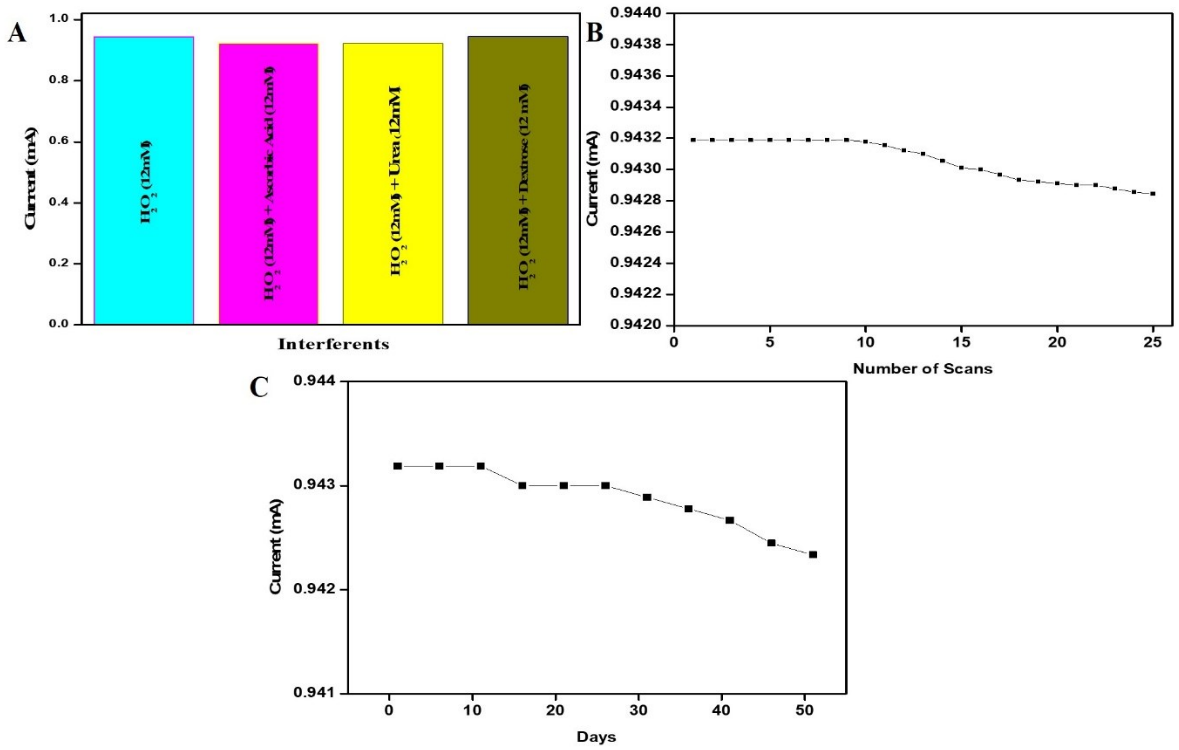

Supp. Figure 6. A) Interference study of pm-CuO/ITO electrode with ascorbic acid (12mM), urea (12mM), and dextrose (12mM) as interferent materials, B) Reproducibility/reusability study of pm-CuO/ITO electrode and **C)** Stability study of pm-CuO/ITO electrode up to 51 days at interval of 5 days.
